# Predicting Crime and Other Uses of Neural Networks in Police Decision Making

**DOI:** 10.3389/fpsyg.2021.587943

**Published:** 2021-10-07

**Authors:** Steven Walczak

**Affiliations:** School of Information and Florida Center for Cybersecurity, University of South Florida, Tampa, FL, United States

**Keywords:** crime, literature review, location, neural network, police, temporal reasoning, geo-spatial reasoning

## Abstract

Neural networks are a machine learning method that excel in solving classification and forecasting problems. They have also been shown to be a useful tool for working with big data oriented environments such as law enforcement. This article reviews and examines existing research on the utilization of neural networks for forecasting crime and other police decision making problem solving. Neural network models to predict specific types of crime using location and time information and to predict a crime’s location when given the crime and time of day are developed to demonstrate the application of neural networks to police decision making. The neural network crime prediction models utilize geo-spatiality to provide immediate information on crimes to enhance law enforcement decision making. The neural network models are able to predict the type of crime being committed 16.4% of the time for 27 different types of crime or 27.1% of the time when similar crimes are grouped into seven categories of crime. The location prediction neural networks are able to predict the zip code location or adjacent location 31.2% of the time.

## Introduction

Crime is a global concern that impacts individuals and society on a daily basis and negatively affects society (Costa, 2010). The ever increasing population along with the rise in urbanization has led to dramatic increases in criminal activities (Fajnzylber et al., 2002; Zhang, 2016), particularly in urban settings (Tumulak and Espinosa, 2017; Stebbins, 2019; Zhu et al., 2019). While increased police presence has been documented to reduce increases in crime (Chalfin and McCrary, 2018), police need to be able to plan for and respond effectively to criminal events, especially those that affect personal or public safety. Police and other forces of civil order would benefit directly from intelligence to help combat all forms of crime and intelligence is a requirement for managing and fighting “intelligent” forms of crime (Tastle, 2013).

Intelligence requires information or data and a methodology for analyzing the data. Law enforcement agencies are an excellent source of large amounts of data. As an example, the number of arrest records available from 2017 to 2019 for the city of Detroit are 81,440, 82,197, and 83,893, respectively, (City of Detroit, 2020) or almost a quarter million records for this 3year period.

Artificial intelligence and more precisely machine learning, provide mechanisms for improving police knowledge of current and potential crimes and facilitating complex anti-criminal decision making. Machine learning techniques scale well to very large amounts of data (Mena, 2011). Neural networks (NNs) are a form of machine learning based on the functionality of the human brain and its neuro-cognitive processing. NNs are the most powerful and accurate clustering technology available (Mena, 2011), enabling high quality information to support police decision making.

This article serves two purposes. First is to assist law enforcement agencies understanding of the capabilities of NNs and the extent of past and current research. A literature review is used to facilitate this knowledge sharing for law enforcement automated decision making and planning researchers and developers. Second is to demonstrate through original research the potential for NNs to be used for analysis of crimes that may facilitate crime preparedness and response by police officers. Existing research as shown through the accompanying literature review focuses on prediction of large areas of generalized increases in criminal activity or the increase in activity for a specific crime. The research presented will be able to predict a specific type of crime as soon as the location and time are known, improving on the larger time horizons of existing research. The other NN model will show that when a type of crime and the time of day is known, a specific area of the city may be immediately identified where that crime is most likely to have occurred, which improves on existing research that uses much larger time horizons. While the research demonstrates that NNs are capable of performing a wide range of crime analyses, they should be viewed as advisory tools or for secondary confirmatory analysis (Tastle, 2013).

## Background on Neural Networks

NNs are a machine learning methodology that excels at classification and prediction problems. Because NNs learn, they are capable of solving arbitrarily complex problems (Hornik, 1991). NNs have been widely applied to solve problems in various domains including business (Vellido et al., 1999), engineering (Bose, 1994), medicine (Baxt, 1991), and the sciences (Sundararaj et al., 1999), and more recently to crime sciences (Oatley et al., 2006), which will be reviewed in the next section.

NNs learn through one of two mechanisms: supervised learning which requires historic data with known results, and unsupervised learning which learns patterns directly from the data. Both techniques require that the NN be trained in order to learn and various training algorithms exist for use in each type of NN learning, with backpropagation being the most popular for supervised learning (Jayalakshmi and Santhakumaran, 2011) and self-organizing maps (SOM being the most prevalent for unsupervised learning; Oatley et al., 2006). Hybrid methods that combine supervised and unsupervised learning also exist, such as the popular convolutional NN (CNN).

**Figure 1:**
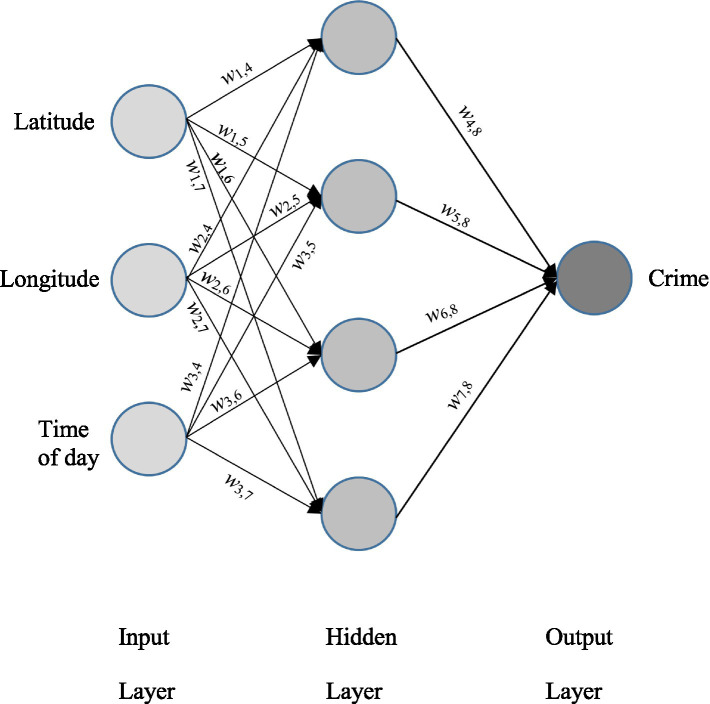
Sample supervised learning ANN [Multilayer Perceptron (MLP)] to predict type of crime from time and place data.

All NNs have an input layer that defines the variables provided to the NN for learning and an output layer that defines the desired classification or prediction solution. Supervised learning and hybrid models also contain one or more hidden layers with each layer being fully connected to the next layer with a weighted connection. Learning occurs in this type of NN by determining the error of a training prediction from the actual value and propagating this error backwards through the network to modify the weights of the connections to better align the prediction with the correct output value. A sample supervised learning NN architecture is shown in Figure 1.

Validation or testing of NN models is done with data that has never been used in the training of the model being evaluated. This is required to eliminate implicit model bias and simulates prospective real-world use of the NN. Typically, available data with known outcomes is divided into a training set and a validation or test set, each with unique examples. Training data should also be adjusted to reflect balanced proportions of the classes to be predicted in order to assist the NN with learning all classes evenly and not overlearning more prevalent classes (Wei and Dunbrack Jr, 2013).

Numerous tutorials exist on how to build and utilize NNs across various domains. The reader is directed to the references for further clarification: Gallo (2015), Jain et al. (1996), Walczak and Cerpa (1999), and Wythoff (1993).

## NNS Used in Crime: Literature Review

An examination of how NNs are being used in Crime Sciences is done by performing a literature review. The purposes of literature reviews may be manyfold and serve to provide an archive and analyses of commonalities across various studies. The current literature review uses the search terms: “neural network” and “crime.” Searches are performed using Google Scholar™ and other library search engines when the documents were not available through Google Scholar™. The review initially considers the top 50 articles returned, excluding patents and citations, without using any date qualifier were examined and the search was re-performed for articles from 2017 to 2020 and the top 25 were examined. Some overlap occurred between the two literature searches. The use of Google Scholar relies on Google Scholar’s relevance ranking for classifying research articles and may be a possible shortcoming of this literature review strategy.

The criteria for the literature review search is to identify NN research be applied to either crime solving or police decision making. The initial search returned 34,800 articles and the recent date search returned 14,000 articles. Four articles meeting the literature review purpose were all from the same book describing different aspects of a single project and so the quantity of articles considered in the initial literature review was extended to the top 55 articles to overcome any bias introduced by this cluster of articles. A total of 68 articles out of the possible 80 that fit the criteria for the current research.

Two categories of NN research were not included, though there exists a plethora of research, because they did not focus on police problem solving and practice. The first category of articles removed is for NNs focused on jurisprudence since these articles are about the application and discovery of relevant laws for legal prosecution, with examples including Bochereau et al. (1991), Fernandes et al. (2019), and Stranieri and Zeleznikow (2012). The other category removed from consideration is NNs applied to cybersecurity, since cybersecurity other than cyber-bullying, is not typically bounded geographically. A few examples of NN research in cybersecurity include Li and He (2020) and Siddiqui et al. (2018).

Kounadi et al. (2020) have recently performed a review and their findings indicate that there is significant growth in spatial crime forecasting research and furthermore claim that the most predominant type of crime forecasting research is the prediction of crime hotspots. Other research has confirmed the emphasis on predicting crime occurrence in specific locations (Loeffler and Flaxman, 2018). The size of the area for which predictions are made varies from study to study. The current literature review presented in this article confirms this with 36 of the 69 (52%) reviewed articles using NNs to predict crime hotspots and shown in Table 1. Neural network predictions from the literature may be for a specific type of crime or the frequency of occurrence of a specific type of crime, or the general location for a specific type of crime. Table 1 defines the type of crime involved in each prediction, the location and size of the area specified for the location, and the type of neural network used to make the prediction. The types of NNs listed in Table 1 are distinguished by the specific type of learning employed in each NN and are defined as:

- Multi-layer perceptron (MLP), a supervised learning feedforward network, most commonly trained using backpropagation or variant thereof;
- Recurrent neural network (RNN), a MLP-like architecture with backward links to previous layers enabling better time series modeling;
- SOM, an unsupervised learning method; and
- Convolutional neural network (CNN), a hybrid unsupervised and supervised learning method that typically contains many hidden layers, frequently used for image classification. This type of NN is frequently referred to as a deep learning neural network.

**Table 1:** NN research predicting crime hot spots.

Type of crime	Type of NN	Location	Area of prediction	References
All	MLP	Various	N/A	Altameem and Amoon, 2019
All	CNN	Rio de Janeiro	Street	Andersson et al., 2017
All	MLP	United States	N/A	Anuar et al., 2015
Motor vehicle accident	MLP	Taiwan	0.1–4.2km	Chang, 2005
All	Supervised learning	City in NE United States	Partial city region	Corcoran et al., 2003
Violent crimes	RNN	Guatemala City	Partial city region	Cortez et al., 2018
Felonies	CNN	New York city	0.18km^2^	Duan et al., 2017
All	CNN	Chicago	Partial city region	Esquivel et al., 2019
All	RNN	San Francisco	City	Feng et al., 2019
Burglary, robbery, assault, and larceny	RNN	New York	Partial city region	Huang et al., 2018
Drug	MLP	London	Neighborhood	Intraligi and Buscema, 2013
12 Crimes	MLP	Poland	N/A	Jankowski et al., 2018
All (aggregate)	Supervised learning	India	Country	Jha et al., 2020
Larceny/theft, assault, other	CNN	San Francisco	Partial city region	Jin et al., 2020
All	CNN	Chicago	Partial city region	Kang and Kang, 2017
Crime around transportation hubs	MLP	Chicago	Transportation hubs	Kouziokas, 2017
All	RNN	Various	N/A	Krishnan et al., 2018
Car theft	CNN	Taoyuan City, Taiwan	PCR	Lin et al., 2018
All	MLP	N/A	N/A	Mao and Du, 2019
All	CNN	Los Angeles	N/A (25 pixels)	Nair and Gopi, 2020
Drugs (crack)	Supervised learning	Pittsburgh	2,150feet^2^	Olligschlaeger, 1997
Assault, robbery, and murder	Supervised learning	Boston	Neighborhood	Patil et al., 2020
Burglary, robbery, and battery		Belgium	200 meter^2^	Rummens et al., 2017
Gang related	Supervised learning	Los Angeles	Partial city region	Seo et al., 2018
All	N/A	Cebu City, Philippines	750–1,000 meters^2^	Tumulak and Espinosa, 2017
All	CNN	Los Angeles	17.8km^2^	Wang et al., 2019
All	RNN,CNN	Los Angeles	Partial city region	Wang et al., 2017a
All	RNN	Chicago	Partial city region	Wang et al., 2017b
All	CNN	San Francisco	Partial city region	Wang et al., 2020
14 Crimes	RNN, CNN	N/A	City	Wawrzyniak et al., 2018
All	CNN	County in China	County	Wu et al., 2018
All	RNN	Chicago and New York	City	Yi et al., 2019
Property crimes	Supervised learning	Boston	Partial city region	Yu et al., 2011
All	MLP	N/A	N/A	Yu et al., 2012
All	RNN	Chicago	N/A	Zhu et al., 2019
All	RNN	Portland	N/A	Zhuang et al., 2017

The length of time in the future that crime forecasts use in making predictions affects how those predictions can be used. Advanced hot spot forecasting times by NNs vary widely from hourly (Wang et al., 2019), to 1day (Corcoran et al., 2003; Duan et al., 2017), to 2weeks (Nair and Gopi, 2020), to 1month (Yu et al., 2011; Cortez et al., 2018; Krishnan et al., 2018; Lin et al., 2018), and even up to 1year (Feng et al., 2019; Yi et al., 2019). Annual predictions are primarily useful for determining adequate budget allocations and performing force recruitment, while hourly and daily future forecasts may help with planning proper responses and re-routing current police resources to hot spot areas.

The second most popular research trend is to utilize NNs for datamining. As stated previously, police generate a large amount of arrest data. Datamining may be used to pull relevant data from natural language police reports, match previous crime data with current crime data to assist investigators, or analyze demographic or spatiotemporal patterns. The datamining analysis of patterns usage of NNs often complements or overlaps with hot spot forecasting, hence there exists some overlap in the reported NN research. Datamining NNs accounted for 23 out of 69 (33%) reviewed crime science NN studies and are displayed in Table 2. The most common NN datamining goal in the previous studies displayed in Table 2 is to cluster similar crimes together from large databases, which may then be used to associate previous crimes with a current or unsolved crime. A secondary use of NN-based datamining is to identify trends in data, which may potentially be used for crime forecasting and preparation.

**Table 2:** NN research performing crime datamining.

Type of crime	Datamining purpose	References
Sex crimes	Determine behaviors and serial behaviors of sex offenders	Adderley and Musgrove, 2001
All	Data mines four databases to determine crime count patterns	Altameem and Amoon, 2019
All	Analyze data to classify crimes into low, medium, or high severity crimes	Anuar et al., 2015
Drugs	Examine police reports to identify related cases	Chau et al., 2002
All	Examine social media posting to identify criminal authors	Chen et al., 2004
All	Examine images on social media to identify ones that could be related to crimes	Chitrakar et al., 2016
Armed robbery	Mine police databases to identify serial crimes	Dahbur and Muscarello, 2003
All	Cluster crime reports into similar categories	Das et al., 2019
Drugs	Examine police notes and databases to determine if drugs were a cause of a crime	Duardo-Sanchez, 2011
Murder (serial)	Identify similarities between crimes in Federal databases	Helbich et al., 2013
Drugs	Identify neighborhood patterns of drug crimes	Intraligi and Buscema, 2013
Four crimes	Looks for trends based on population and other census factors across 6,100 United States cities	Kaikhah and Doddameti, 2006
Burglary	Identify similarities between previous crimes and determine if any similarity to a current crime	Keyvanpour et al., 2011
All	Identify a specific gun from bullet evidence	Kou and Tung, 1994
14 Crimes	Identify seasonality and location trends	Li et al., 2010
Drugs	Evaluate ethnicity effects in drug crimes	Massini, 2013
Murder	Classify murders and identify serial patterns	Memon and Mehboob, 2003
Burglary	Link known offenders to unsolved crimes	Oatley and Ewart, 2003
Burglary	Link known offenders to unsolved crimes	Oatley et al., 2006
Assault, robbery, murder	Determine hour of day and day of week patterns	Patil et al., 2020
Burglary, battery, and robbery	Match behavior evidence of a crime to known criminals	Rummens et al., 2017
All	Examine relations between demographics and crimes	Wu et al., 2018
All	Identify objects in crime scene images	Zhang et al., 2020

Prediction of criminal recidivism, or the likelihood that a criminal will either repeat an offense or be arrested for a different more serious offense, is another popular area of NN research. NNs have been identified as one of the most popular datamining techniques applied to criminal recidivism over the last two decades (Hashim and Nohuddin, 2018). A total of six articles out of 69 (9%) articles reported NN applications to predict recidivism for various crimes (Babcock and Cooper, 2019) or races (Jain et al., 2019) or general prediction of recidivism (Caulkins et al., 1996; Palocsay et al., 2000; Liu et al., 2011; Chun et al., 2019). Prediction of likely recidivists is normally used to improve rehabilitation, but may also be used to identify possible crime perpetrators.

The final area of NN research that will be discussed is NN research meant to facilitate police investigative work and police response. NNs are a recommended tool for making criminal investigation recommendations and providing confirmatory analysis (Tastle, 2013). A datamining example that fits into this classification of NN applications in crime science and police decision making is the work by Kou and Tung (1994) who tried to identify a specific gun, as opposed to the type of gun, based on the markings on a bullet. This study is originally listed with the datamining NNs due to its need to analyze data from numerous databases containing specific gun-bullet markings. Other investigation decision support examples are: a NN that identifies what the type of glass is, out of a possible seven types, from a small piece of glass shard or fragment found at a crime scene (El-Khatib et al., 2019), and a NN that identifies blood or other threatening objects in crime scene photos (Nakib et al., 2018). Strano (2004) describes how to develop a NN to assist investigators in performing deductive murder profiling.

Similar to investigative support, but more directly affecting police response, NN models have been developed to assist police in identifying the likelihood of a type of crime being committed. Buscema and Intraligi (2013) report on a NN that attempts to distinguish crack based drug crimes from other types of drug crimes. To assist officers responding to an unknown crime type, a NN identifies likely crimes based on the image of a building or street scene (Fu et al., 2018). The last NN reported in this literature review is a NN that classifies anonymous tips from police hot lines into one of 20 possible categories of crime (Pinho et al., 2017), though the crime type may have been mentioned in the anonymous tip.

The NN models reviewed in this section are composed of both supervised learning (*N*=35) and unsupervised learning (*N*=29) NN models. The count of NN models trained using unsupervised learning methods includes the CNN hybrid method. The quantity of each learning method used in prior literature indicates that neither method is dominant, but supervised learning is used slightly more frequently. Nineteen of the 35 supervised learning examples used MLP or a derivative thereof. The difference between the type of training used for the various NN models may have to do with the type of problem being modeled. SOM and CNN NNs typically are used in other domains for image or audio classification, while supervised learning methods are used for classification and forecasting problems, and supervised learning RNN models are used for problems that are based on time, such as time-series forecasting.

The review of existing literature has shown that while NN research in criminal science is expanding, with the focus of existing research being on staffing or budget planning months or even years in the future. The research presented in this article examines utilization of data that could be collected from an initial crime report (*via* 911 in the United States or 112 in the European Union) to provide either type of crime or crime location information immediately to emergency responders. The intended immediate use of NN predictions from a minimal amount of information, distinguishes the presented research from prior research.

## NN Models to Predict Crime Type and Crime Location

To demonstrate NN model usage in police crime predicting and preparedness, two NN prediction models are developed. Both models utilize supervised learning NN architectures. All NN models are developed using NeuralWare’s® Professional II Plus® neural network shell tool.

### Data and Method

Data for the NN models were obtained from the open access RMS Crime Incidents crime data available from the city of Detroit Michigan (City of Detroit, 2020). A total 272,623 records were available at the time the research models were created. The records as grouped by Detroit criminal charge number are clustered into 38 different crimes, which are spread across 30 zip code regions of the city. A zip code is a postal code used to define geographic regions for postal delivery. The crime codes associated with each cluster are defined in the Appendix. Other data included a date-time stamp, the day of the week, the hour of the day, and the longitude and latitude of the incident. The data covered a period of just over 4years from 2016 to the first quarter, 2020.

For all NN models described, a variety of architectures are attempted, and we follow the NN research guidelines of Walczak and Cerpa (1999) and Zhang (2007), including using one or two hidden layers and varying the number of NN nodes within each hidden layer. The number of nodes in any hidden layer starts at one half the number of nodes in the previous layer and is increased incrementally until performance starts to drop. This method helps to prevent over-learning which can decrease resultant generalization performance. The incremental increase for the first hidden layer is two nodes at a time and if a second hidden layer is used, then that layer is increased a single node at a time for all possible first hidden layer architectures.

Two different training methods are used: backpropagation and radial basis function (RBF). All NN models are trained for a minimum of 150,000 training epochs and training stops when the root mean squared error becomes less than 0.05 or remains constant for 1,000 training epochs. Backpropagation is used because it is the most common NN training method and thus enables better cross study comparisons. Additionally, the backpropagation learning algorithm has been shown to be a reliable universal classifier (Hornik, 1991). RBF training sometimes outperforms backpropagation when extrapolation from data is requires (as opposed to interpolation) or when data is more limited (Walczak and Cerpa, 1999). RBF NN require an initial hidden layer that utilizes a radial basis function as the transfer function, which effectively determines the proximity of an input value to a desire output value. While a large number of data examples are available for training and validation/testing, the limited number of variables used as input (independent variables) for the NN models may impose data limitations and hence the desire to also utilize RBF trained NN models. The best performing architecture of all attempted NN architectures is reported for each NN crime forecasting problem.

### NN to Predict Type of Crime

The first NN model’s purpose is to predict what crime is occurring when given only location and time information. This is to simulate a 911 call response when the caller is unable to describe the crime that is occurring. It may also be viewed as a preparatory model for police officers as they are assigned to different neighborhoods on a specific day of the week and time and consequently hear a call for help. The goal of the NN is to predict the most likely crime that is occurring in order to better prepare police response.

Data were originally divided evenly into a training set and a validation set, with extra examples due to an odd number of samples for a specific crime cluster placed in the validation set. Backpropagation trained NNs with both a single hidden layer and two hidden layers, which would allow for greater nonlinearities in the solution surface (Walczak and Cerpa, 1999), are developed. The predictions from all of these NNs however only correctly classified crimes from 3 to 4 of the 38 possible crime clusters. This is due to the very large difference in the quantities of any type of crime compared to others. For example, crimes clustered as assault crimes had 71,931 total cases, whereas the harassment and stalking crime cluster only had 41 total cases. Over represented clusters in the training data is a cause of poor classification and prediction performance by machine learning methods including NNs (Lin et al., 2013).

To compensate for the severe imbalance between the crime cluster representations, the data is pre-balanced so that only clusters that have at least 600 total samples are used, which reduces the number of clusters from 38 to 27 [removing the crime clusters for minor in possession of alcohol (*N*=10), animal cruelty (*N*=3), extortion (*N*=147), gambling (*N*=6), harassing and stalking (*N*=41), health and safety (*N*=30), invasion of privacy (*N*=34), liquor violations (*N*=340), personal protection order (*N*=6), probation violation (*N*=8), non-sex solicitation (*N*=25)]. From the remaining 27 clusters, a sample of 350 crime reports were used unless the total cases for that cluster was less than 700, then half of the cases were used. To add a very small influence for crime clusters with a very large number of cases a proportional number of extra crime samples for those clusters was added to the training set to a maximum of 370 samples for any single crime cluster. This created a training set with 9,503 training samples approximately evenly distributed between all 27 crime type clusters. The remaining 262,473 crime report samples served as the validation set for the fully trained NN. It should be noted that no validation sample is ever used for training the NN and is therefore representative of real-world prospective use of the NN.

The input variables for the NN models consisted of the day of the week and the hour of the day and one of three variables for location. Location variables could be either a categorical variable representing the 30 different sip codes within Detroit, or the longitude and latitude (LL), or a combination of both zip code and LL. The output vector for all NN models is a 27 variable set, one for each of the 27 crime clusters. The output of the NN is evaluated with respect to both prediction accuracy and cluster coverage, to avoid cases of overfitting on a small number of clusters with large sample size. Backpropagation trained NNs outperformed the RBF NN models. The best performing NN for each of these evaluation measurements is reported along with the best performing RBF model since it reflects a middle position.

### NN to Predict Crime Location

The second NN model’s purpose is to predict the location of a crime when only the general type of crime (i.e., cluster) and time of day and day of week are known. This simulates the situation when an emergency call comes in and the crime reporter, victim or otherwise, is unsure of their location. Such could occur if the reporter were a visitor to the city or was in some other way unable to determine their location, such as a kidnap victim in the trunk of a vehicle. The goal of this NN is to predict the most likely zip code. The choice of the zip code area for crime location prediction follows the common practice reported by other hot spot crime estimation NNs as shown in Table 1, where an area of prediction (e.g., city) is used instead of spot identification of a precise geographic location (i.e., longitude and latitude).

Both the NN to predict a crime given the location and time and the one to predict the zip code region of a crime given the crime and time focus on spatiotemporal relationships. Both NNs are ambitious in attempting to predict a specific crime happening at a specific time or a generalized location of a crime. A more precise location prediction is not attempted since prior research has shown that there is no basis for short term crime forecasting due to evidence from data showing that spatial heterogeneity and time lag cannot accurately be reflected in short-term prediction (Zhu et al., 2019).

The input values for the crime location prediction NN consists of a 27 value categorical variable for the crime cluster being reported and the day and time. The output consists of 30 variables, one representing each of the 30 zip codes present in the city of Detroit. Again, both backpropagation and RBF training are used, but the backpropagation NN models outperform the RBF models. Since location is important, and the boundaries of zip code areas are irregular, in addition to reporting exact matches to the zip code area, a fuzzy algorithm is implemented to identify when the NN predicted zip code location is contiguous with the actual zip code of the location, meaning that if the predicted and actual zip code locations share a border, then a near miss is recorded. The NN with the highest exact match as well as the highest near miss prediction percentage are reported.

## Results and Discussion

The crime type NN prediction models performed best when using the zip code only for location information. The results are displayed in Table 3. The two-hidden layer MLP had 12 nodes in its first layer and four nodes in its second layer. The single-hidden layer MLP had 12 nodes in its hidden layer. Lastly the RBF NN had an association layer of 54 nodes and a hidden layer of 12 nodes.

**Table 3:** NN prediction of crime type results (27 crime clusters).

NN model	Crime prediction accuracy (%)	Number of crime clusters predicted
Two-hidden layer backpropagation trained MLP	16.4	11
Single-hidden layer backpropagation trained MLP	7.9	24
RBF trained NN	12.9	17

Results for the NN models predicting the location, by zip code, are displayed in Table 4. The two-hidden layer MLP NN had 27 nodes in its first layer and six nodes in its second layer, while the single-hidden layer MLP NN had 36 nodes in its hidden layer.

**Table 4:** NN prediction of crime location (30 zip code regions).

NN model	Exact location prediction (%)	Within 1 (near miss and exact) location prediction (%)
Two-hidden layer MLP	8.2	28.4
Single-hidden layer MLP	7.6	31.2

Recall that for both sets of results, the prediction accuracies are over 262,799 reported crime arrests. This means for the NN that predicted 16.4% of the crimes when given just the zip code, time of day, and day of week, it was able to predict over 43,100 specific crime clusters. However, methods to improve this performance should be investigated. Much of the hot spot NN prediction research shown in Table 1, when not predicting a hot spot for all crimes, predicts hot spots likelihood for only a subset of all possible crimes, from 1 to 12 types of crime. If the number of crimes predicted is limited to five or six crimes, with both burglary with forced entry and without forced entry combined into the single cluster burglary, this would cover from 72.3 to 79.2% of all reported crimes. However, only evaluating the top six crimes, even though it covers almost 80% of the crimes, is still not useful to police, since they would need to know when the other 31 types of crimes are being committed.

Therefore, a NN model is developed using the top six clusters with a seventh variable representing all other types of crime. The variables are balanced as before with approximately 1,000 samples per cluster for a total of 7,607 training samples and all other data used for validation/testing with 265,016 crime samples. The same research process is duplicated for the new NN crime prediction model as used for the original crime prediction NN. Results for the new seven cluster NN crime prediction models are displayed in Table 5, which shows a 65% overall increase in performance at the six cluster level predicting over 27% of crimes and over a 45% increase in performance at the seven cluster prediction level predicting almost 24% of crimes across all crime clusters. This indicates that trying to be too precise concerning which crime causes some classification problems with NNs. This may be because the principle crime could be classified as many different types of crime or a crime may be misclassified with respect t the degree or severity of the crime. It is beneficial for police to be able to have advanced knowledge if a crime is violent or non-violent and future research will further examine this distinction to create new sets of clusters for prediction based on the presence of violence in a criminal situation.

**Table 5:** NN prediction of crime type for largest 6 clusters and other (clusters=7).

NN model	Crime prediction accuracy (%)	Number of crime clusters predicted
Single hidden layer MLP	27.1	6
Single-hidden layer MLP	23.8	7

Knowing the location of a crime before the actual location is determined can help police response time for these type of cases. The NN model given only the crime cluster, time of day, and day of week is able to determine the approximate location, within one contiguous zip code area (out of 30) almost one third of the time. Faster response times may improve the chance of catching the criminal and preserving crime scene evidence.

Predicting the location of a crime could be combined with other NN models performing hotspot predictions to determine when it is likely that certain types of crimes are more likely to occur and then the location NN would be able to tell the general vicinity of the crime almost. Although the reported research used the 30 zip code regions to define location and proximity, other measurements could also be used, but it should be kept in mind that as the regions get smaller, this introduces a greater degree of error due to the larger number of locations that must be correlated to the different crimes. Square regions that are X blocks by Y blocks could serve as an alternate location prediction paradigm, which would eliminate need to map police response to zip code regions.

Recall that prior research has shown that there is no theoretical evidence to support short term forecasting of crimes, including both the crime and location (Zhu et al., 2019). This is why the majority of the research reported in Table 1 predicted hot spots from 1day to 1year in the future. To further examine this claim, a key is created for all of the original Detroit crime data for the years 2017–2020, a total of 284,869 crime records. The keys were then searched to identify duplicate records. Only 3.4% of all crimes were repeated at the same location on the same day and at the same hour, which left 275,192 total crimes without repeats for these 39months. Only the hit-and-run, disorderly conduct, and homicide clusters had repeat offenses at greater than 1%. For the 27 clusters defined for the crime prediction NN, nine of these clusters had no similar offenses, defined as a repeat of a crime cluster offense at the same location and at the same day and hour, but for different months and years. This does not account specifically for repeat victimization (Farrell and Sousa, 2001), since the day of week and hour of day must also match. Similar crimes with regard to geospatial and temporal data does not play a role in facilitating crime or location predictions, since there was only a 1.26% duplication between the training data and the test/validation data. The duplicate crime analysis confirms that trying to predict crimes based solely on geospatial and temporal information is problematic due to the very low repetition of crimes with similar time and location.

However, the NN models reported in this paper do just that: predicting a crime or location at the immediate time of occurrence. This is an advancement over traditional hot spot predictions which are normally for crimes overall and for much longer periods of time in the future.

## Summary and Conclusion

The utilization of machine learning in general and NNs in particular in crime sciences is both prevalent and expanding. Traditional NN research in crime science focuses on future hot spot predictions, datamining large corpora of data to connect relevant information together regarding a crime or serial crime or criminals, determining the probability of criminal recidivism, and analysis of crime scene objects and data to assist with criminal investigations. A small literature review of these four types of NN research in crime science is presented, focusing on more recent research. NNs have additionally been used to help identify cybercrime activities and also in a datamining sense to identify relevant laws and decisions for jurisprudence. Both of these utilizations of NNs have not been reviewed as the focus for this article is on police practice.

Machine learning enables the automatic identification of complex and usually nonlinear associations between crimes and geospatial and temporal factors of those crimes, which are beyond the scope of traditional parametric statistical approaches. These associations further enable systems that can then predict the type of crime or crime category and also the probable location of a crime as demonstrated in the NN examples presented in this article.

The only data used for these NNs are the general type of crime, time of day, and day of the week. The other data item available to each NN model depends on what is being predicted with the zip code of occurrence as an independent variable for predicting the dependent crime cluster or the independent crime cluster variable for predicting the dependent location.

NN models are presented that can predict a specific crime cluster 16.4% of the time over 27 crime clusters or if the number of clusters is limited to seven to better focus on the most prevalent crimes, then a prediction accuracy of 27.1% is achieved. This prediction accuracy is significantly better than just randomly guessing, which would produce an estimated prediction accuracy of 14.3% with a value of *p*<0.001 (standard *Z*-Test). This type of on-time knowledge of the type of a potential crime may increase police response preparedness.

Another NN model identifies the location of a crime within one neighboring zip code, when the location is not immediately known, 31.2% of the time. This result indicates that specific crime types are at least partially geographically clustered.

These NN models show an advancement over current NN research that is more limited to forecasting overall crime for a much larger period and further into the future. Future research is needed to further investigate the use of NNs, possibly using additional temporal or different sized geospatial cues to improve near-term (immediate) crime predictions.

## Data Availability Statement

Publicly available datasets were analyzed in this study. This data can be found at: https://data.detroitmi.gov/datasets/0825badfe6304620a998d162be0e135e_0/data?geometry=-83.503%2C42.264%2C-82.695%2C42.442.

## Author Contributions

The author confirms being the sole contributor of this work and has approved it for publication.

## Conflict of Interest

The author declares that the research was conducted in the absence of any commercial or financial relationships that could be construed as a potential conflict of interest.

## Publisher’s Note

All claims expressed in this article are solely those of the authors and do not necessarily represent those of their affiliated organizations, or those of the publisher, the editors and the reviewers. Any product that may be evaluated in this article, or claim that may be made by its manufacturer, is not guaranteed or endorsed by the publisher.
