# Supplementary material for: Predicting Crime and Other Uses of Neural Networks in Police Decision Making
Source: Front Psychol. 2021 Oct 7;12:587943. doi: 10.3389/fpsyg.2021.587943 (PMC8529125; doi:10.3389/fpsyg.2021.587943)
Supplement: Supplementary file 1 [file Table_1.DOCX]

Supplementary Material

# Supplemental Appendix 1

The clustering approach utilized to define crime type clusters for the reported research utilizes several criteria including: number of unclustered crime samples, crime description and similarities in arrest codes (first 3 digits). The clusters names and their corresponding Detroit RMS arrest codes are displayed in Table A.1. If a cluster only had a single incident it was removed from the data, which reduced the initial clusters from 42 to 38.

**Supplemental Table A.1. Crime clusters based on Detroit RMS arrest codes**

| **Cluster** | **Number of incidents** | **Arrest codes contained in cluster** |
| --- | --- | --- |
| Alcohol - minor in possession | 10 | 41096 |
| Animal Cruelty | 3 | 55086 |
| Arson | 2600 | 20000. 20001, 20098 |
| Assault | 71931 | 13001, 13002, 13012, 13096 |
| Burglary - entry no force/intent | 2824 | 22002, 22044, 22055 |
| Burglary - entry with force | 21792 | 22001, 22004, 22022, 22033, 22075 |
| Child/family neglect | 2055 | 38001-38003, 38006 |
| Contributing to the delinquency of minor | 1 | 38005 |
| Cybercrime | 1 | 29097 |
| Damage to Property | 32791 | 29000-29002 |
| Disorderly conduct | 1113 | 53001, 53002, 53012 |
| Drugs | 7169 | 35001, 35002, 35010, 35012, 35032, 35060, 35062 |
| Entry w/o permission | 668 | 22003 |
| Extortion | 147 | 21000 |
| Forgery/counterfeiting | 925 | 25000, 25003 |
| Fraud | 19239 | 26001-26006, 26099, 30001, 48072 |
| Gambling | 6 | 39001, 39003 |
| Harassing | 41 | 13080, 13084, 53009 |
| Health & Safety | 30 | 55000 |
| Hit and run | 2506 | 54001 |
| Homicide | 892 | 9001-9004, 13071 |
| Intimidation/Stalking | 2978 | 13003, 13016, 13081, 13082 |
| Invasion of privacy | 34 | 57002 |
| Kidnapping | 658 | 10001, 10002 |
| Larceny | 43480 | 23001-23007, 23009, 23010, 23079 |
| Liquor violation | 340 | 41002 |
| Lost & found | 1 | 9915 |
| Miscellaneous | 1918 | 73000 |
| Motor Vehicle - Stolen | 24549 | 24001-24003, 24008, 24011 |
| Obstruction | 5025 | 48000, 48001, 50000 |
| Personal Protection order | 6 | 50070 |
| Probation | 8 | 50011, 50012 |
| Prostitution | 2532 | 400001, 400002 |
| Runaway | 1311 | 70000, 70070 |
| Sex Crime 1 | 1914 | 11001, 11003, 11005 |
| Sex Crime 2 | 1549 | 11007, 11008 |
| Sex Crime 3, 4, and other | 1193 | 11002, 11004, 36001-36005 |
| Solicitation - non-sex | 25 | 75000 |
| Theft | 11930 | 12000-12002, 12009, 12070, 28000, 28002, 28003, 30002 |
| Tobacco - minor | 1 | 55070 |
| Trespass | 787 | 57001 |
| Weapon/gun | 5644 | 52001-52003, 52013, 52022, 52095 |
